# “Transcranial direct current stimulation for chronic foot pain: A comprehensive review”

**DOI:** 10.1016/j.ensci.2024.100498

**Published:** 2024-03-20

**Authors:** Roberto Tedeschi

**Affiliations:** Department of Biomedical and Neuromotor Sciences, Alma Mater Studiorum, University of Bologna, Bologna, Italy

**Keywords:** Transcranial direct current stimulation (tDCS), Chronic foot pain, Plantar fasciitis, Neuromodulation, Pain management

## Abstract

**Background:**

Chronic foot pain, including conditions such as plantar fasciitis, presents a significant challenge to patients and healthcare providers. Traditional treatments often offer limited relief, prompting exploration of alternative therapies. Transcranial direct current stimulation (tDCS) has emerged as a noninvasive brain stimulation technique with potential for alleviating chronic pain syndromes.

**Methods:**

A review was conducted following the JBI methodology and adhering to PRISMA guidelines. Searches were performed in databases including MEDLINE, Cochrane Central, Scopus, and PEDro, supplemented by grey literature sources and expert consultations. Studies were included if they investigated tDCS as an intervention for chronic foot pain, assessed its efficacy, safety, or mechanisms of action, and were published in English.

**Results:**

A total of three papers were included in the review. The findings indicate that tDCS holds promise for managing chronic foot pain, including plantar fasciitis. Main results suggest significant reductions in pain intensity and improvements in related outcomes following tDCS treatment.

**Conclusions:**

This review underscores the potential of tDCS as an alternative therapy for severe lower-extremity pain, highlighting the need for further research to optimize its parameters and long-term effects. tDCS emerges as a promising neuromodulation approach for chronic foot pain management, offering insights for enhancing patient outcomes and quality of life.

## Highlights

• Anodal tDCS reduces chronic foot pain intensity.
• tDCS improves pain-related anxiety in plantar fasciitis.
• Promising adjunctive therapy for treatment-resistant cases.

## 1 Introduction

Chronic foot pain, including conditions such as plantar fasciitis, poses significant challenges for patients and healthcare providers alike [1,2]. Traditional treatment approaches often provide limited relief, leading to a search for alternative therapeutic options [[3], [4], [5], [6], [7], [8]]. In recent years, transcranial direct current stimulation (tDCS) has gained attention as a noninvasive brain stimulation technique with the potential to modulate pain perception and provide relief for various chronic pain syndromes [[9], [10], [11]]. tDCS involves the application of low-intensity electrical currents to specific areas of the brain, resulting in the modulation of neuronal activity. By targeting the primary motor cortex and related cortical areas, tDCS can potentially influence pain processing pathways and alleviate chronic foot pain [12]. Several studies have explored the use of tDCS in the management of chronic pain, yielding promising results [12,13]. However, there is still a need for a comprehensive review of the existing literature on tDCS for chronic foot pain, specifically focusing on plantar fasciitis. This review aims to provide an overview of the current evidence regarding the efficacy and mechanisms of action of tDCS in the management of chronic foot pain [[14], [15], [16]]. The findings from this review and case study may contribute to a better understanding of the role of tDCS in chronic foot pain management, highlight potential areas for further research [17], and inform healthcare providers and patients about the potential benefits and limitations of tDCS as an alternative therapeutic approach [[18], [19], [20], [21], [22]]. By exploring the existing literature and presenting a clinical case, this review aims to shed light on the utility of tDCS as a promising intervention for chronic foot pain, providing insights that may aid in improving patient outcomes and quality of life [23,24]. This scoping review aimed to provide a comprehensive overview of the existing literature on the use of transcranial direct current stimulation (tDCS) for the management of chronic foot pain. Specifically, the review sought to identify and summarize relevant studies investigating the efficacy, safety, and potential mechanisms of action of tDCS in the context of chronic foot pain.

## 2 Methods

The present scoping review was conducted following the Joanna Briggs Institute (JBI)methodology [25]for scoping reviews. The Preferred Reporting Items for Systematic reviews and Meta-Analyses extension for Scoping Reviews (PRISMA-ScR) [26] Checklist for reporting was used.

### 2.1 Research team

To support robust and clinically relevant results, the research team included authors with expertise in evidence synthesis, quantitative and qualitative research methodology, sport and musculoskeletal rehabilitation.

### 2.2 Review question

We formulated the following research question: “What is the current evidence regarding the use of transcranial direct current stimulation (tDCS) for the management of chronic foot pain?”

### 2.3 Eligibility criteria

Studies were eligible for inclusion if they met the following Population, Concept, and Context (PCC) criteria.

Population:

• Participants with chronic foot pain or conditions leading to chronic foot pain, such as plantar fasciitis, neuropathy, or arthritis.

Concept:

• Studies investigating the use of transcranial direct current stimulation (tDCS) as an intervention for chronic foot pain.
• Studies assessing the efficacy, safety, and potential mechanisms of action of tDCS in managing chronic foot pain.
• Studies examining the effects of tDCS on pain intensity, functional outcomes, quality of life, or other relevant outcomes in individuals with chronic foot pain.

Context:

• Studies conducted in any setting, including clinical or research settings.
• Studies published in English language.
• Studies with available full-text articles.
• Studies published from inception to the present

### 2.4 Exclusion criteria

Studies that did not meet the specific PCC criteria were excluded.

### 2.5 Search strategy

An initial limited search of MEDLINE was performed through the PubMed interface to identify articles on the topic and then the index terms used to describe the articles were used to develop a comprehensive search strategy for MEDLINE. The search strategy, which included all identified keywords and index terms, was adapted for use in Cochrane Central, Scopus, PEDro. In addition, grey literature (e.g. Google Scholar, direct contacts with experts in the field) and reference lists of all relevant studies were also searched. Searches were conducted on 23 May 2023 with no date limitation.

### 2.6 Study selection

After completing the search strategy, the search results were collected and imported into EndNote V.X9 (Clarivate Analytics). To ensure the accuracy of the dataset, duplicates were removed using the EndNote deduplicator, resulting in a file containing a unique set of records. This file was then made available to the reviewers for further processing. The selection process involved two levels of screening using the Rayyan QCRI online software. At the first level, titled “title and abstract screening,” two authors independently reviewed the articles based on their titles and abstracts. Any conflicts or discrepancies between the reviewers' decisions were resolved by a third author. The goal of this level was to assess the relevance of each article to the research question based on the provided information. The second level of screening, known as “full-text selection,” also involved two authors independently reviewing the full texts of the selected articles. The purpose of this level was to assess the eligibility of each article based on its complete content. Again, any conflicts or disagreements between the reviewers were resolved through discussion and, if necessary, consultation with a third author. Throughout the selection process, detailed records were maintained, documenting the reasons for excluding articles that did not meet the inclusion criteria. This documentation followed the latest published version of the Preferred Reporting Items for Systematic Reviews and Meta-analyses (PRISMA 2020) flow diagram. The PRISMA flow diagram visually represents the screening process, indicating the number of articles identified, screened, assessed for eligibility, and included in the final analysis. By adhering to these rigorous selection procedures and reporting guidelines, transparency and reliability were ensured in the article selection process, enabling a comprehensive and systematic approach to be taken in the scoping review.

### 2.7 Data extraction and data synthesis

Data extraction was conducted using a pre-designed data extraction form, specifically developed for this scoping review. The form was created based on the JBI (Joanna Briggs Institute) data extraction tool, tailored to capture key information from the selected articles. The extracted data included the following details: authors, country of publication, year of publication, study design, patient characteristics, pertinent findings or outcomes, type of intervention, related procedures, and any relevant additional information. Descriptive analyses were performed on the extracted data to summarize the characteristics of the included studies. The results were presented in a numerical format, using frequencies and percentages to report the studies identified and included in the scoping review. This approach allowed for a concise representation of the distribution and composition of the included studies. The description of the search decision process, including the number of articles identified, screened, assessed for eligibility, and ultimately included in the review, was systematically mapped. This mapping process provides transparency and clarity in documenting the selection process, allowing for a comprehensive understanding of the article selection flow. Importantly, the extracted data were summarized in tabular form, presenting the main characteristics of the included studies. These summary tables provide a structured overview of the key information extracted from each study, facilitating comparison and analysis of the findings across the included articles. Overall, the presentation of the extracted data in this scoping review primarily relies on concise and informative summary tables, providing a clear and organized representation of the main characteristics and results of the included studies.

## 3 Results

**Fig. 1:**
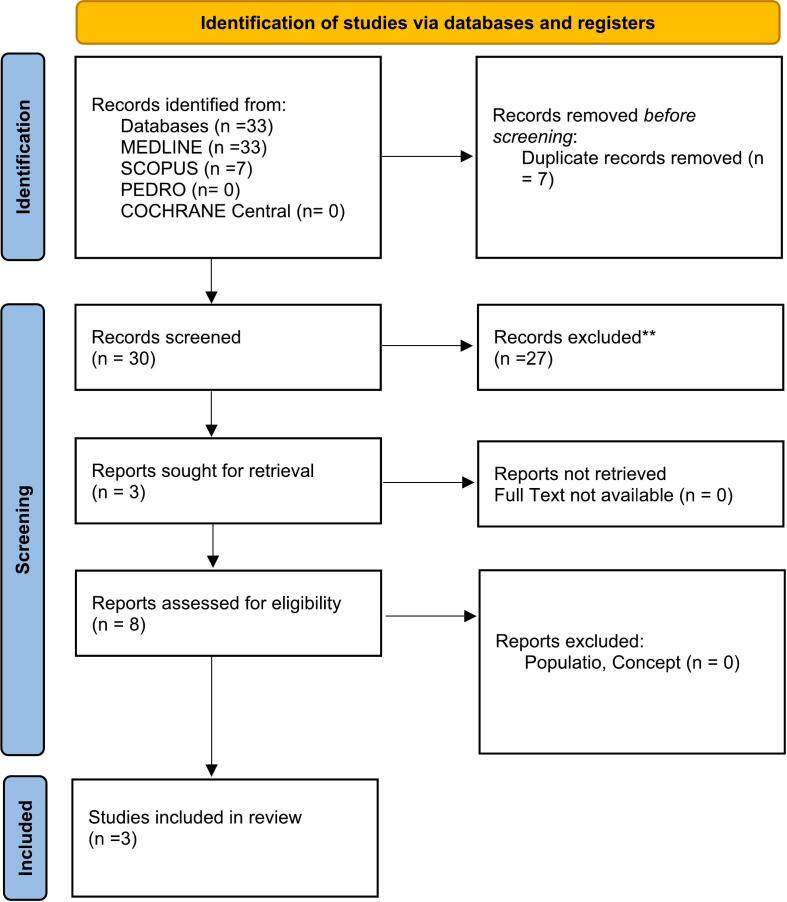
Preferred reporting items for systematic reviews and meta-analyses 2020 (PRISMA) flow-diagram. The figure illustrates the flow of studies through the systematic review process according to the PRISMA guidelines (2020).

As presented in the PRISMA 2020-flow diagram (Fig. 1), from 33 records identified by the initial literature searches, 30 were excluded and 3 articles were included. (See Table 1, Table 2.)

**Table 1:** Main characteristics of included studies.

N°	AUTHOR	TITLE	YEAR	COUNTRY	STUDY DESIGN
1	Mohomad et al. [27]	Severe chronic heel pain in a diabetic patient with plantar fasciitis successfully treated through transcranial direct current stimulation	2015	Tunisia	Case Report
2	Concerto et al. [28]	Anodal transcranial direct current stimulation for chronic pain in the elderly: a pilot study	2016	Usa	Trial
3	Lerma-Lara et al. [29]	Transcranial direct-current stimulation (tDCS) in the primary motor cortex and its effects on sensorimotor function: a quasi-experimental single-blind sham-controlled trial	2021	Spain	Trial

tDCS: Transcranial Direct Current Stimulation.

**Table 2:** Types of interventions.

Population	Method	Outcome
- A 65-year-old diabetic man with treatment-resistant right heel pain due to plantar fasciitis.	- The patient underwent five tDCS treatment sessions on 5 consecutive days. - Each session involved 20 min of anodal tDCS applied over the left primary motor cortex leg area.	- The neurostimulation protocol resulted in a significant decrease in pain intensity. - Pain-related anxiety also showed a significant reduction following tDCS treatment. - The effects of tDCS treatment on pain intensity and pain-related anxiety persisted beyond the stimulation period, lasting up to 1 week. - The patient discontinued the use of opioid medication after receiving tDCS treatment. - Therapeutic neuromodulation with tDCS appears to be a promising alternative for the management of severe lower-extremity pain, specifically in the case of treatment-resistant plantar fasciitis.
- Ten patients with chronic plantar fasciitis and symptomatic treatment resistance were enrolled in the study.	- Visual Analog Scale (VAS) was used to assess pain intensity. - The Foot Function Index (FFI) was used to measure foot function. - The Pain Anxiety Symptom Scale (PASS-20) was employed to evaluate pain-related anxiety symptoms. - The Hamilton Rating Scale for Depression (HDRS-17 items) was used to assess depression symptoms. - Anodal tDCS treatment was administered over the motor area of the leg contralateral to the symptomatic foot for 20 min, at 2 mA, for 5 consecutive days. - Pre-tDCS (T0), post-tDCS (T1), 1 week (T2), and 4 weeks (T3) post-treatment assessments were conducted.	- Anodal tDCS treatment resulted in a significant improvement in pain intensity. - FFI scores showed a significant improvement post-treatment, which was maintained up to 4 weeks. - PASS scores demonstrated a significant improvement following treatment and remained improved at the 4-week follow-up. - Anodal tDCS treatment did not report specific results related to depression symptoms. - Patients reported a reduction in the consumption of pain medication tablets following anodal tDCS treatment. - The results suggest that anodal tDCS treatment targeting the primary motor cortex may be an effective option for reducing chronic foot pain and improving pain-related anxiety in elderly patients with treatment-resistant plantar fasciitis.
- A total of 100 healthy individuals were included in the study.	The sham-tDCS (s-tDCS) group consisted of the remaining 50 participants who received sham stimulation. - Two-point discrimination (2-PD) test, tactile acuity threshold, pressure pain threshold (PPT), and electromyographic (EMG) activity were assessed. - Assessments were conducted before and after the tDCS application. - EMG activity during maximal voluntary contraction in the biceps brachii and rectus femoris was measured. - The active-tDCS (a-tDCS) group consisted of 50 participants who received 2 mA of anodal stimulation in the primary motor cortex (M1) for 20 min.	- No significant between-group differences were observed in any of the sensorimotor variables assessed. - Within the a-tDCS group, there were significant pre- and post-intervention differences in tactile acuity threshold in the thenar eminence of the hand (*p* = .012, d = 0.20). - Significant pre- and post-intervention differences were also found in the PPT of the rectus femoris (*p* = .001, d = − 0.17) within the a-tDCS group. - Although no between-group differences were observed, within-group differences in EMG activity were statistically significant for the biceps brachii (*p* = .023, d = − 0.16) and the rectus femoris (*p* = .011, d = − 0.14) within the a-tDCS group. - The study showed no significant between-group differences in sensorimotor outcomes. - A single session of tDCS in isolation produced immediate but very small effects on sensorimotor function in healthy participants.

a-tDCS: Active Transcranial Direct Current Stimulation, EMG: Electromyographic, FFI: Foot Function Index, HDRS-17: Hamilton Rating Scale for Depression (17 items), M1: Primary Motor Cortex, PASS-20: Pain Anxiety Symptom Scale (20 items), PPT: Pressure Pain Threshold, s-tDCS: Sham Transcranial Direct Current Stimulation, tDCS: Transcranial Direct Current Stimulation, VAS: Visual Analog Scale.

## 4 Discussion

The present study explored the potential of transcranial direct current stimulation (tDCS) as a promising treatment modality for chronic foot pain, specifically in patients with plantar fasciitis. The findings demonstrated that anodal tDCS targeting the primary motor cortex of the leg contralateral to the affected foot led to significant improvements in pain intensity, foot function, and pain-related anxiety. These positive effects were sustained up to 4 weeks post-treatment. The observed reduction in pain intensity aligns with previous studies highlighting the analgesic effects of tDCS in various chronic pain conditions. Adams et al., 2024 [30] conducted a systematic review with meta-analysis on tDCS for orthopedic pain, highlighting its potential efficacy. Rahimi et al., 2023 [31] focused on tDCS in knee osteoarthritis patients, suggesting its efficacy in this population. Additionally, Lloyd et al., 2020 [32] conducted a systematic review and meta-analysis on tDCS for fibromyalgia pain, further supporting the analgesic effects of tDCS in chronic pain conditions. These studies collectively contribute to the growing body of evidence supporting the use of tDCS as a promising intervention for managing chronic pain, including foot pain associated with conditions like plantar fasciitis. However, more research is warranted to establish tDCS's efficacy, optimal parameters, and mechanisms of action in diverse patient populations.The primary motor cortex is a key region involved in pain processing and modulation, and by modulating its activity using tDCS, pain perception can be altered. This study adds to the growing body of evidence supporting the efficacy of tDCS in pain management [33]. Furthermore, the improvement in foot function, as measured by the Foot Function Index (FFI), suggests that tDCS may have a positive impact on functional outcomes related to plantar fasciitis. This finding is particularly important as foot pain can significantly impair mobility [[34], [35], [36]] and quality of life in affected individuals. By enhancing foot function, tDCS may contribute to better overall physical functioning and increased engagement in daily activities. The alleviation of pain-related anxiety observed in this study is another notable outcome. Chronic pain often coexists with psychological distress, and addressing both pain and associated psychological comorbidities is crucial for comprehensive pain management [12]. The Pain Anxiety Symptom Scale (PASS-20) [37] was utilized to assess anxiety symptoms specifically related to pain, and the significant reduction observed suggests that tDCS may have a broader impact on psychological well-being in patients with chronic foot pain. Importantly, the sustained effects of tDCS up to 4 weeks post-treatment indicate the potential for long-term benefits. This finding suggests that a short course of tDCS sessions may have a lasting impact on pain relief and functional improvement in patients with chronic foot pain. Such long-lasting effects are valuable in reducing the reliance on pain medications and improving overall treatment outcomes. It is worth noting that this study focused on a single case, and larger controlled trials are needed to further validate the effectiveness of tDCS in treating plantar fasciitis and similar conditions. Additionally, the specific mechanisms underlying the observed effects of tDCS in foot pain management warrant further investigation [38,39]. In conclusion, this study provides promising evidence for the use of anodal tDCS as a potential treatment option for severe lower-extremity pain, such as chronic foot pain due to plantar fasciitis. The observed reductions in pain intensity, improvements in foot function, and alleviation of pain-related anxiety highlight the potential of tDCS as a non-invasive and well-tolerated intervention. Further research in larger populations is warranted to confirm these findings and optimize the parameters of tDCS for maximum therapeutic benefit in patients with chronic foot pain [40].

### 4.1 Strengths and limitations

#### 4.1.1 Strengths

The study explored the potential of tDCS as a treatment modality for chronic foot pain, specifically in patients with plantar fasciitis, addressing an area with limited treatment options.

Significant improvements were observed in pain intensity, foot function, and pain-related anxiety, suggesting the potential of tDCS to address multiple aspects of chronic foot pain.

The sustained effects of tDCS up to 4 weeks post-treatment indicate the potential for long-term benefits, reducing the reliance on pain medications and improving overall treatment outcomes.

The study utilized validated assessment tools, including the Foot Function Index (FFI) and the Pain Anxiety Symptom Scale (PASS-20), to comprehensively evaluate treatment outcomes.

The study adds to the growing body of evidence supporting the efficacy of tDCS in pain management, particularly in chronic pain conditions.

#### 4.1.2 Limitations

The specific mechanisms underlying the observed effects of tDCS in foot pain management were not fully elucidated. Further research is needed to explore these mechanisms.

While significant improvements were observed, the study did not report on potential adverse effects or safety concerns associated with tDCS treatment.

The study did not include a control group for comparison, which may introduce bias and limit the ability to draw definitive conclusions about the efficacy of tDCS.

The duration of follow-up was limited to 4 weeks post-treatment, and longer-term outcomes beyond this timeframe were not assessed. Future studies should consider longer follow-up periods to evaluate the durability of treatment effects.

In clinical practice, transcranial direct current stimulation (tDCS) offers a promising avenue for managing chronic foot pain, particularly in patients with conditions like plantar fasciitis. Healthcare providers can integrate tDCS into treatment protocols for patients who have not responded adequately to traditional therapies. Patient selection is key, focusing on those with chronic foot pain resistant to conventional treatments. Assessments using validated tools gauge pain intensity, foot function, and psychological factors to tailor tDCS protocols to individual needs. Before treatment, ensure patients are well informed about tDCS benefits, risks, and limitations. Administer consecutive daily tDCS sessions following established protocols, monitoring treatment response regularly and adjusting as needed. Combining tDCS with other therapies may enhance overall efficacy, and maintaining thorough documentation ensures continuity of care. By adopting tDCS in clinical practice, healthcare providers can offer an additional avenue for managing chronic foot pain and improving patient outcomes.

## 5 Conclusions

In conclusion, this study highlights the potential of anodal transcranial direct current stimulation (tDCS) as a promising adjunctive treatment for chronic foot pain, particularly in patients with conditions such as plantar fasciitis. The results demonstrate significant reductions in pain intensity and improvements in pain-related anxiety, suggesting tDCS as a valuable therapeutic option for individuals resistant to conventional therapies or experiencing high levels of pain-related anxiety. However, further research is warranted to validate these findings, refine treatment protocols, and evaluate long-term efficacy.

## Ethics approval and consent to participate

Not applicable.

## Consent for publication

Not applicable.

## Availability of data and materials

Not applicable.

## Funding

No Funding.

## CRediT authorship contribution statement

**Roberto Tedeschi:** Writing – original draft, Supervision, Methodology, Investigation, Conceptualization.

## Declaration of competing interest

The authors declare that there are no conflict of interests. This study did not receive any specific subsidies from public, commercial or non-profit funding agencies. The authors are doctoral students, university professors, and clinicians with no economic or financial interests.
